# Ranging behaviour and habitat preferences of the Martial Eagle: Implications for the conservation of a declining apex predator

**DOI:** 10.1371/journal.pone.0173956

**Published:** 2017-03-17

**Authors:** Rowen van Eeden, D. Philip Whitfield, Andre Botha, Arjun Amar

**Affiliations:** 1 DST-NRF Centre of Excellence Percy FitzPatrick Institute of African Ornithology, Department of Biological Sciences, University of Cape Town, Cape Town, South Africa; 2 Natural Research, Banchory Business Centre, Burn O’Bennie Road, Banchory, Aberdeenshire, United Kingdom; 3 Birds of Prey Programme, Endangered Wildlife Trust, Modderfontein, South Africa; University of Lleida, SPAIN

## Abstract

Understanding the ranging behaviours of species can be helpful in effective conservation planning. However, for many species that are rare, occur at low densities, or occupy challenging environments, this information is often lacking. The Martial Eagle (*Polemaetus bellicosus*) is a low density apex predator declining in both non-protected and protected areas in southern Africa, and little is known about its ranging behaviour. We use GPS tags fitted to Martial Eagles (n = 8) in Kruger National Park (KNP), South Africa to describe their ranging behaviour and habitat preference. This represents the first time that such movements have been quantified in adult Martial Eagles. Territorial eagles (n = 6) held home ranges averaging ca. 108 km^2^. Home range estimates were similar to expectations based on inter-nest distances, and these large home range sizes could constrain the carrying capacity of even the largest conservation areas. Two tagged individuals classed as adults on plumage apparently did not hold a territory, and accordingly ranged more widely (ca. 44,000 km^2^), and beyond KNP boundaries as floaters. Another two territorial individuals abandoned their territories and joined the ‘floater’ population, and so ranged widely after leaving their territories. These unexpected movements after territory abandonment could indicate underlying environmental degradation. Relatively high mortality of these wide-ranging ‘floaters’ due to anthropogenic causes (three of four) raises further concerns for the species’ persistence. Habitat preference models suggested Martial Eagles used areas preferentially that were closer to rivers, had higher tree cover, and were classed as dense bush rather than open bush or grassland. These results can be used by conservation managers to help guide actions to preserve breeding Martial Eagles at an appropriate spatial scale.

## Introduction

Understanding an animal’s behaviour, such as its movement and habitat utilization, is increasingly seen as important in species conservation [1]. Ranging behaviours can inform managers about the most important habitats required for species preservation, and furthermore allow managers to conceptualize the scales at which conservation strategies should be implemented [2]. However, these behavioural data may not be readily available for species of concern [3]. For instance species that inhabit remote and challenging environments, e.g. seabirds foraging in open oceans [4], or those that are wide ranging, e.g. migratory species [5] may suffer from a lack of data needed to fully realize their conservation.

Improvements in remote tracking technology have improved the ability to accurately understand species’ ranging behaviours and to understand their habitat preference [6]. This has been particularly important for species that were traditionally considered difficult to study or found within challenging landscapes [2, 6]. For highly mobile species, modern GPS devices have enabled fine-scale tracking, providing insights into their life histories that were previously poorly understood [5, 7–9]. Historical methods of animal tracking, such as radio tracking or mark recapture, were often prone to location inaccuracy, observer effects, or observation bias [10–12].

Novel insights on movement behaviour gained through improvements in technology can improve the ability to inform species conservation. For instance, by modelling the flight heights and ranging behaviour of GPS tagged Bearded Vulture (*Gypaetus barbatus)* Reid et al. [13] made recommendations on wind farm placements to minimize the likelihood of collisions with turbines. Fine scale mapping of home range use can also refine estimates of carrying capacities, facilitate understanding of species’ resource requirements, or assess and predict impacts of human activities [6, 14].

The Martial Eagle (*Polemaetus bellicosus*) has declined throughout much of its sub-Saharan African range, and is now listed as globally Vulnerable [15]. Within South Africa, large declines have also been recently detected [16]. Cloete [17] found reporting rates between the two Southern African Bird Atlas Projects [18] declined by up to 60% over the last 20 years. Worryingly, these declines were also recorded in South Africa’s large protected areas [17, 19], with declines of 54% recorded for Kruger National Park (KNP), which has long been regarded as a stronghold for this species in the region [20]. The specific causes driving these declines have yet to be established although threats to the species include persecution, habitat transformation, electrocutions and drowning in farm reservoirs [21–25].

The habitat use and ranging behaviour of Martial Eagles is poorly understood. The species occurs at low breeding densities with inter-nest distances averaging ca. 12 km in KNP [20], and so is predicted to have very large home ranges [26]. Investigations regarding nest site selection along power lines in the Karoo region of South Africa suggested that Martial Eagles prefer areas that are dominated by shrub land and avoid cultivated landscapes, and prefer to nest in areas with irregular terrain [27]. However, apart from this study, there are no other published studies on the habitat preferences of this species.

In this paper, we describe the first study to examine individuals’ ranging behaviour and habitat use for this threatened species, using GPS tracking devices. GPS tags were fitted to adult Martial Eagles within KNP and tracked over the course of three years. Our objective was to provide baseline information on this species’ ranging behaviour and its habitat preference. We aimed to estimate home range size, and how this changes through the breeding cycle. We investigated habitat preference (in relation to topography, land cover type, tree cover, rivers, and roads) of our tracked birds within their territories both in the breeding and non-breeding periods.

## Methods

### Study area

**Fig 1:**
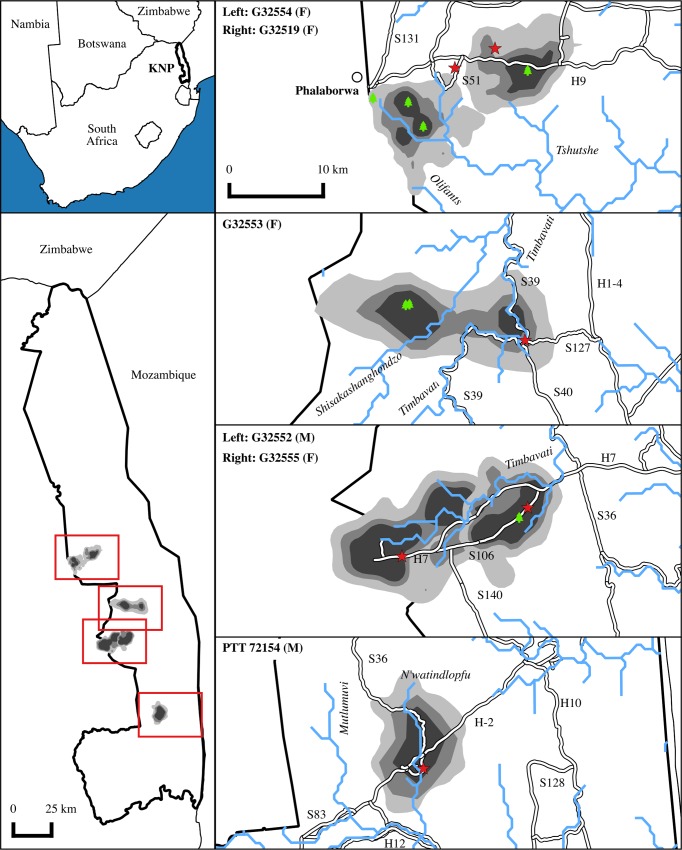
Home range estimators (50, 75, 95% Kernel Density Estimates) for six of eight adult Martial Eagles that were classed as territorial. Eagles were GPS tracked in KNP, South Africa (top left). Home ranges are shown in relation to KNP borders. Expanded plots of home ranges are shown (right) in relation to the KNP boundary (thick black line), main rivers (blue lines e.g. Timbavati), roads (thin parallel black lines e.g. S39, nest sites (green trees), and capture locations (red stars). Panels on the right showing home ranges are set to the same scale as given in the upper right panel.

KNP is South Africa’s largest protected area covering ca. 20,000 km^2^ and the flagship South African National Park (SANParks). KNP forms the eastern border of the country with Mozambique to the east and Zimbabwe to the north (Fig 1). KNP lies within the savannah biome [28] and habitat types vary greatly across the Park supporting diverse biotic compositions in different regions [29]. The Park is divided by geology into basalts in the east while granites dominate the bedrock to the west. Rivers align with geological structures, and as such, typically occur at higher densities on the western granites compared to the eastern basalts [30]. Underlying geology also tends to favour greater tree cover (vegetation >5 m) on the western granites compared to basalts [31]. KNP is relatively low lying and flat with elevation in the Park varying from ca. 200–840 m above mean sea level (asl).

### Study species

The Martial Eagle is Africa’s largest eagle species [32] with females averaging 4.7 kg (3.9–5.3 kg) and males averaging 3.3 kg (2.2–5.1 kg) [33]. Although Martial Eagles have a widespread distribution, they are sparsely distributed, with an estimated 800 mature individuals in South Africa, Lesotho and Swaziland [34]. Martial Eagles reach sexual maturity at six years when they also moult into adult plumage that is distinct from that of juveniles and sub-adults [35]. Martial Eagles are tree nesting raptors, which are bi-parental and, likely, typically monogamous, and are thought to hold large territories throughout their adult lives [20, 35, 36]. Breeding typically occurs every second year laying a single egg, with incubation lasting 47–51 days, the fledging period lasting 90–109 days [35]. Martial Eagles occur in the highest densities in the savannah biome [34]. They are present in a wide range of habitats including open shrublands with tree cover near rivers, and open farmlands where there are trees or pylons for nesting [18, 27, 32–34]. Birds are nearly absent from mountainous regions and grasslands void of tree cover [34].

### Ethical statement

The trapping method used in this research is an internationally recognised and ethical procedure [37] carried out under relevant permits and licenses from local authorities: The research was approved by South African National Parks Animal Use and Care Committee (Reference number 13–5); University of Cape Town Science Faculty Animal Ethics Committee (Approval number: 2013/V7/AA); Department of Environmental Affairs and Tourism: Threatened or Protected Species (ToPS) (Permit number WM 1297/2013).

### Tracking data and mortality estimation

Six adult Martial Eagles were trapped between late July and early August 2013, and a further two individuals were trapped in March 2016 using a bal-chatri [38] containing small chickens (*Gallus gallus domesticus*) and fitted with 70 g GPS satellite tags (PTT 100, Microwave Telemetry, Columbia, Maryland, U.S.A.). GPS units were fitted using a backpack-mounted harness made from 0.55” Teflon® Ribbon (Bally Ribbon Mills, Pennsylvania, U.S.A). For this study, only adult eagles (> 6 years old), which are easily classified as such from plumage characters, were opportunistically selected for capture when they were found perched along tourist roads in KNP. Birds were sexed based on mass, which in cases involving breeding birds was verified from behaviour at the nest site. In addition to a GPS tag, birds were fitted with alphanumerically unique 26 mm stainless steel rings (SAFRING authority card 12956). GPS tags recorded hourly positions, accurate to ±18 m (http://microwavetelemetry.com). These fixes were obtained between 5:00 and 17:00 during winter months (starting 21 April) and between 5:00 and 18:00 during summer months (starting 03 September). Daily tracking data was received every three days and the data inspected when received. Efforts were made to recover tags that were no longer moving. The causes for this (e.g. mortality) were deduced as far as possible and reported in context in the results section.

### Ranging behaviour and home range estimation

Within raptor populations adults can take on the role of either territory holders or more wide-ranging floaters [39]. Although Martial Eagles are typically thought to hold territories from adulthood [35], it is unknown in the literature if a section of the adult population range more widely. Therefore we first defined the movement strategy for each individual using net squared displacement (NSD) and a latent state model using the ‘lsmnsd’ package [40] in R [41]. NSD measures the squared distance between each location and the first location, and when plotted over time provides insight into specific movement strategies e.g. migration (cyclic departure and return to and from the same geographic space), dispersal (departure from one geographic space to another), nomadism (in raptor biology population floaters often move in a way that can be described as nomadic), and resident (in raptor biology termed territorial) [42]. The NSD plots were also visually inspected and compared to those in Bunnefeld et al. [42] to ensure correct classification.

To investigate ranging behaviour and calculate home range sizes we used the adehabitatHR package in R to estimate Minimum Convex Polygons (MCP) and Kernel Density Estimates (KDE) of the species Utilization Distribution (UD) [43]. UDs are the most common method employed in visualizing and calculating home ranges [44, 45], and more generally express the traditional concept of a home range [46]. UDs were calculated using the *h*_ref_ method (grid = 100 m, or if the grid size was too small to allow estimation the grid size was increased by 50 m increments until the estimation was made). We calculated 95, 75 and 50% utilization distributions to map the areas used during general home range use for all individuals in QGIS [47]. Furthermore we estimated a 100% MCP which encloses all GPS fixes in the smallest possible convex polygon and as such also includes fixes that may be atypical to an individual’s predominant home range. MCPs have been used historically for radio tracking studies on Martial Eagles [36] and we therefore calculated these to enable comparisons with these historical findings.

To explore whether home range sizes changed in relation to the different stages of breeding (non-breeding vs. breeding period, as determined by nest checks) we calculated monthly home range sizes. For individuals tracked over more than one year we calculated annual home range sizes using 95% utilization distributions. For individuals that had more than one movement behaviour (e.g. territorial and floater–see Results), home range sizes were calculated separately for each behaviour.

Lastly to investigate ranging behaviour further we assessed individuals’ movement step lengths (distance moved between hourly locations). We compared their movements between months to assess seasonal effects, the breeding period and non-breeding period, and different movement strategies (territorial and floater). This comparison was made using a generalised linear mixed model in R package lme4 [41, 48]. The distance between points (step lengths) was the dependent variable and individual ID was fitted as a random effect. To calculate the step lengths we measured the straight-line distance between successive hourly GPS locations between 6:00 and 18:00. Where more than one hour elapsed between two GPS locations, the distance travelled between these hours was excluded so that only hourly movements were conserved for analysis. Models were assessed using AICc scores calculated during model comparison in the R package MuMIn [49]. If two or more candidate models were within 4 AICc, then we performed model averaging on these candidate models.

### Habitat preference: Environmental variables

We explored habitat preference using a number of environmental variables (S1 Fig). To describe topographic influences (elevation and slope) we used a 90 m digital elevation model [50]. Tree cover preferences were investigated using a 30 m resolution continuous fields of tree cover map that describes the proportion of vegetation >5 m in height [51]. The importance of rivers was inferred using a 1:50 000 resolution HydroSHEDS river network layer [52] that has global coverage. As roads are known to influence hydrology, tree height and species assemblages [53] we incorporated a roads layer into our environmental dataset. Lastly, we included a recent (2013/2014) 72 class South African national land cover dataset [54] that categorizes South Africa into land classes at a 30 m resolution (S1 Table). Categories include vegetation type e.g. grassland, open bush, dense bush, and anthropogenic categories such as urban development.

### Habitat preference: Statistical analyses

Habitat preference of Martial Eagles during the non-breeding period was investigated by calculating the likelihood of occurrences of GPS tracking fixes and a set of randomly distributed points which fell within each individuals 95% KDE to describe the use of the environmental variables described above [55] (data available in supporting information S1 File). This is the same approach used by Reid et al. [13] to model Bearded Vulture habitat use that were fitted with identical tags programmed with the same duty cycle of hourly fixes. Data from the breeding period was excluded from this specific model because birds under different constraints (e.g. breeding vs. non-breeding) can effect the interpretation of habitat preference models [56]. Similarly, for birds that vacated their territories (n = 2), the data associated with their non-territorial movements were excluded from the analyses. The presence/pseudo-absence of Martial Eagles was modelled as a function of the elevation, slope, % tree cover, and land cover class at each presence/pseudo-absence location, and the distance from each presence/pseudo-absence location to the nearest road, and river. In addition we included the distance to the edge of the 95% KDE territory to account for territorial behaviour (data available in supporting information S2 File). The probability of occurrence in relation to these habitat variables was modelled using binomial generalised linear mixed-effects models.

We then examined habitat preference during the breeding period in a separate model. A 100 m buffer was placed around nest sites, and all points (both presence and pseudo-absences) falling within this buffer were excluded, to ensure fixes biased towards the nest e.g. during incubation were not over represented in the analyses. Because only three individuals bred during the study, the breeding period habitat preference was modelled using a generalised linear model, with individual ID fitted as a fixed effect. In this second model we fitted distance to the nest site as an additional explanatory variable.

The separation between breeding and non-breeding periods was made accurately by examining nest centric behaviour; the onset and cessation of daily visits of the tagged bird to within 500 m of the nesting site described the start and end of each individuals’ breeding season.

Numerical variables were centred and standardised: $V_{2}=\frac{\left(V_{1}-\bar{x}\right)}{s(V_{1})}$, where V_1_ is the unstandardised variable, $\bar{x}$ is the mean of V_1_, and *s* is the standard deviation of V_1._ For factor data (e.g. national land cover), categories that contained less than 2% of the total data (e.g. “permanent water” which classifies water bodies such as lakes) were grouped and assigned into a category “other” (S1 Table). The random points (pseudo-absences) were generated using QGIS [47]. Three times the number of random points to real bird fixes were generated for each bird.

Model selection was based on the model with the lowest corrected Akaike Information Criteria (AICc) using the MuMIn package in R to compare all possible model combinations. If models were within 4 AICc then we model averaged the top model candidates within 4 AICc to get averaged fixed effects estimates and calculated the fixed effects confidence intervals (lower: 2.5%, upper: 97.5%) using MuMIn.

Model fit was assessed using receiver operator curves (ROC) in R package ROCR [57]. ROC assess the predicted classification of absences and presences into their correct categories and the area under curve (AUC) was thus used to determine model performance; values over 0.9 are typically associated with an accurate model and AUCs of 0.7–0.9 categorize models with moderate predictive power, while models with an AUC <0.7 are generally considered to have relatively poor predictive power [58]. Correlation between independent variables was tested for, and we used a correlation coefficient of 0.3 as a cut off for assessing relationships between variables.

## Results

**Fig 2:**
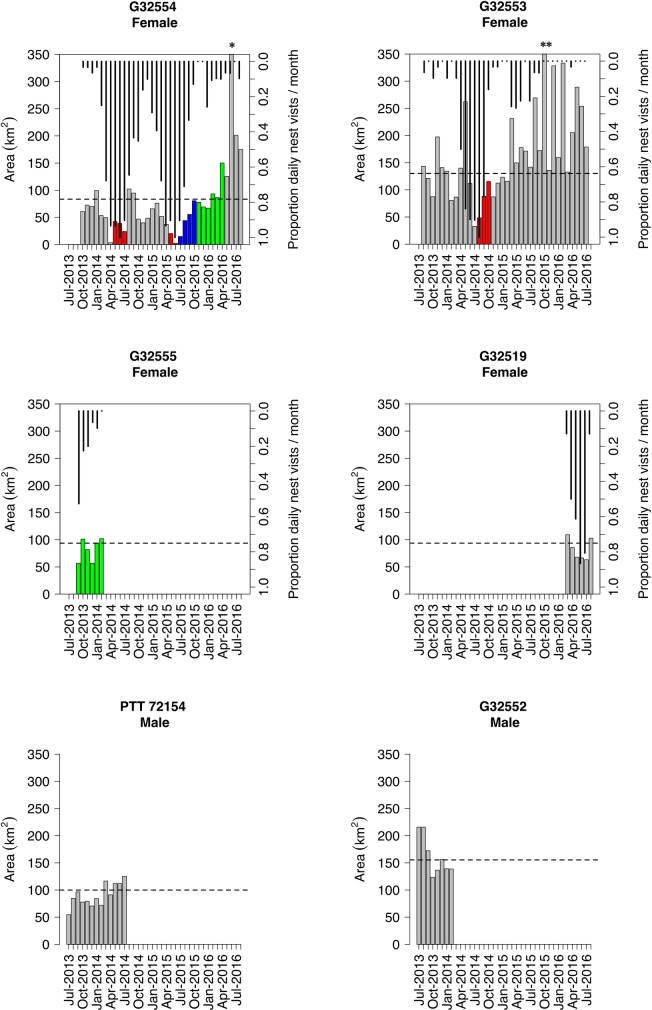
The 95% kernel density estimate (KDE) home range sizes for each individual by month. Missing bars indicate no data due to cessation of tag transmission. Coloured bars indicate months in which the individual bird was recorded to incubate (red), raise a fledgling (blue) or provision a fledged chick in post fledging dependency (green). Horizontal dashed lines indicate the 95% KDE for the entire dataset of each individual. Vertical lines indicate the proportion of days per month that a nest site was visited by the individual. * 1493 km^2^; ** 2319 km^2^

Individual Martial Eagles were tracked for a mean duration of 479 ± 374 days (range = 160–973 days; Table 1). We tracked six females and two males. Three female individuals (G32554, G32519, and G32553) were tracked through at least one breeding cycle (Fig 2). Another female (G32555) was tagged whilst she was still provisioning a fledged chick. The remaining two females (G32551 and G32516) and the two tracked males (PTT 72154 and G32552) did not have known nest sites, nor did their tracking data indicate an obvious nesting location.

**Table 1:** Tracking data summary for adult Martial Eagles GPS tracked in KNP. The attributed causes of transmission cessation are shown where applicable; devices still transmitting at the time of publication are represented by NA.

Individual	Sex	Dates tracked	# Days	# GPS locations	Transmission cessation (cause)
G32554	F	09/10/2013–21/09/2016	1078	11758	NA
G32553	F	26/08/2013–22/09/2016	1123	14874	NA
G32551	F	01/08/2013–06/07/2014	339	4064	Hunted
G32555	F	12/09/2013–19/02/2014	160	2358	Conspecific conflict
G32519	F	16/03/2016–22/09/2016	187	2673	NA
G32516	F	22/03/2016–20/05/2016	59	869	Hunted
G32552	M	23/08/2013–07/02/2014	168	2532	Conspecific conflict
PTT72154	M	30/07/2013–20/08/2014	386	5466	Electrocution

### Home ranges

Six individuals were classed as territory holders and appeared to remain in spatially confined regions or home ranges for most of their time (Fig 1 and S2 Fig). The 95% kernel density estimated home range size for these individuals averaged 108.42 ± 29.51 km^2^ (Table 2). Home range sizes for two individuals tracked over multiple years differed between years with an increasing home range size trend from 2013 to 2016 (G32554: 2013 = 69.12 km^2^, 2014 = 71.69 km^2^, 2015 = 74.7 km^2^, 2016 = 107.33 km^2^; G32553: 2013 = 137.87 km^2^, 2014 = 110.86 km^2^, 2015 = 281.84 km^2^, 2016 = 204.96 km^2^).

**Table 2:** Home range estimations (Minimum Convex Polygon: MCP and Kernel Density Estimators: KDE) for each adult Martial Eagle tracked in the KNP showing the extent of areas used (km^2^) under different GPS location densities (50–95%).

Individual	Sex	MCP 100%	KDE 95%	KDE 75%	KDE 50%
Territorial					
G32554	F	489.55	83.49	31.47	11.56
G32553	F	4 279.37	185.12	80.48	37.56
G32555	F	316.91	93.78	49.21	26.00
G32519	F	415.31	85.47	37.13	14.01
G32552	M	174.40	157.90	86.39	45.48
PTT72154	M	155.40	99.83	50.89	24.35
Floater					
G32551	F	46 860.2	43 052.44	19 812.69	8 781.842
G32516	F	23 368.65	45 337.43	19 012.47	7 103.56
G32553	F	22 943.05	10 830.8	2 503.894	1 048.142
PTT72154	M	713.3828	-	-	-

**Fig 3:**
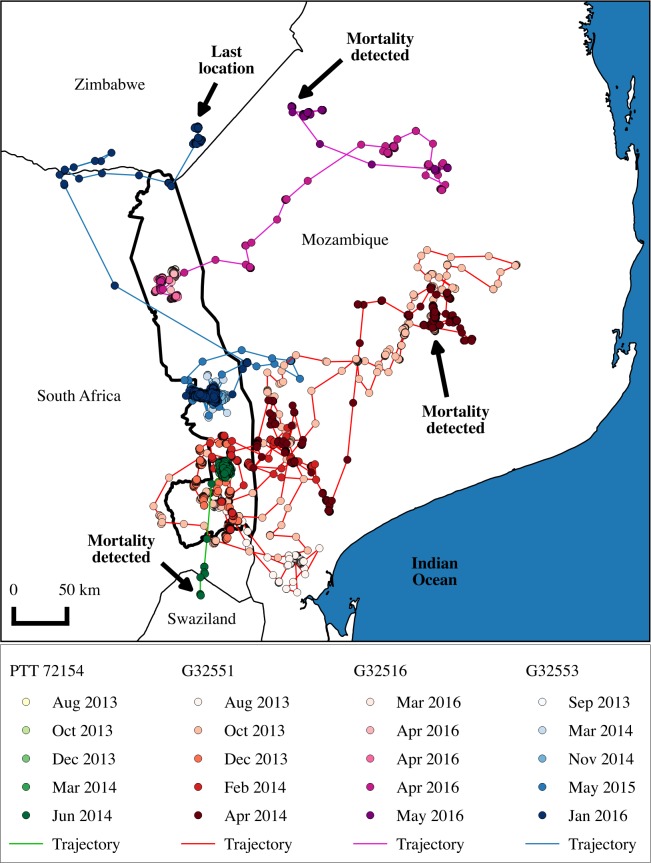
Movements of floater adult Martial Eagles (G32551 and G32516), and those that were territorial but vacated their territories (PTT 72154 and G32553) from KNP, showing the area covered during tracking. GPS locations are coloured by month to visualize movements through time (see bottom legend). The location of each bird’s death, or last known location, is also indicated.

Another two females (G32551 and G32516) did not remain on a territory and were classed as floaters; these individuals ranged widely covering a 95% KDE area of 44,194.93 ± 1615.73 km^2^ before they were both hunted in Mozambique (Fig 3; Table 2). Although our sample size (n = 2) was too small for any formal comparison, visual inspection of monthly home range sizes indicated that female home ranges reduced in size prior to and during incubation. These monthly home ranges increased during the nestling period, and when raising a chick or provisioning for a fledged juvenile they were similar to the average home range size (Fig 2). There did not appear to be any large differences in home ranges sizes between the sexes, although male home ranges were slightly larger than females (95% KDE; males: 128.86 ± 41.06 km^2^, females: 98.21 ± 21.71 km^2^).

MCPs were often 1–35 times larger than 95% KDEs (Table 2). Visual inspections of GPS locations relative to MCPs and KDEs suggest individuals with much large MCPs made temporary movements beyond their normal range (S3 Fig), and this explained the larger home range sizes estimated from these MCPs.

Two individuals (G32553 and PTT 72154) left their territories without returning. PTT 72154 made a long ranged movement into Swaziland where it was likely electrocuted shortly afterwards. G32553 vacated her territory and moved north into Zimbabwe after 1087 days of tracking, in the previous 2 years she had been unsuccessful in rearing any young and failed on incubation during the first year.

**Fig 4:**
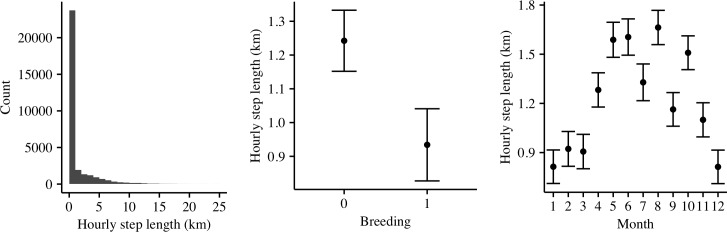
Frequency of step lengths in 1 km (0–10 km) and 15 km (10–100 km) intervals (left), and fixed effects plots from a generalised linear mixed model investigating step lengths between the breeding and non-breeding period (centre) and between months to assess seasonality (right).

Hourly step lengths in Martial Eagles were generally small; ca. 57% of hourly movements were less than 200 m apart (Fig 4). Step lengths were smaller in the breeding-period compared to the non-breeding period and tended to increase in winter months compared to the summer months (S2 Table; Fig 4). There were no clear differences between the step lengths of territorial birds or floaters.

### Habitat preference

We examined habitat preference during the breeding and non-breeding period separately. therefore we used data from three individuals during months when these individuals showed nest centric behaviour typical with the pre-incubation, incubation, and the nestling period (G32554, G32553, G32519; S3 Table). Data from the two individuals whose movements were not confined to a stable home range (G32551 and G32516) were also excluded from analyses, as we were concerned with calculating territorial habitat preferences only. As such when another two individuals (G32553 and PTT 72154) abandoned their territories we excluded these data when not holding a territory.

**Fig 5:**
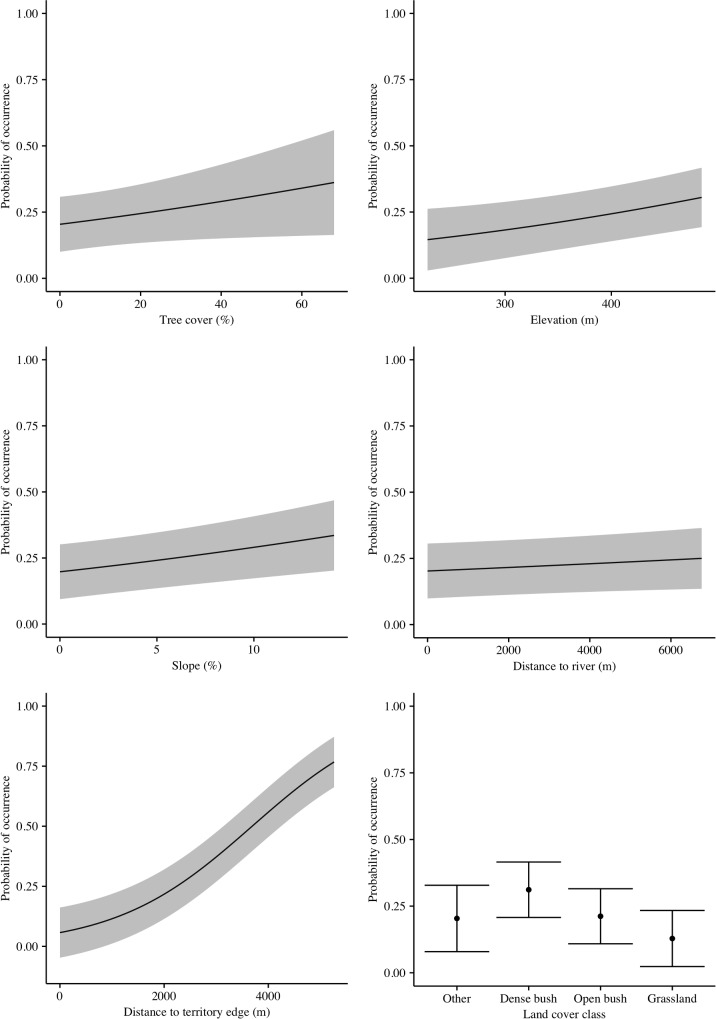
Generalized linear mixed model (GLMM) predictive fixed-effects plots showing the modelled habitat utilization of territorial Martial Eagles tracked after their capture in KNP according to several habitat features: tree cover, elevation, topographic slope, distance to nearest river, distance to nearest road, and land cover. Solid lines show the predicted relationships, with 95% CL captured within grey shaded areas. The bottom right panel shows the predicted probability for the categorical factor, land cover, with 95% confidence limits represented by vertical lines.

Habitat preference by territorial adults during the non-breeding period was associated with all of our environmental variables, with our top model incorporating all terms (S4 Table). However the next best model (which excluded distance to roads) was within 4 AICc and therefore we model averaged the top two models. The average model indicated that habitat preference was associated with higher percentage tree cover, areas that were farther from main rivers, at higher lying elevations, on steeper slopes, and areas that were farther from the territory edge (Table 3, Fig 5). Because the confidence intervals for the distance to roads variable overlapped zero, we cannot attribute this variable to having any influence over the species habitat preference. Additionally, dense bush areas tended to be selected over areas comprising of open bush, grassland, or other habitat types (Table 3, Fig 5).

**Table 3:** GLMM results showing the model averaged fixed effects (scaled) estimates used to predict the occurrence of Martial Eagles during the non-breeding period and the results from the general linear model investigating breeding period habitat preference. The models included, tree cover, elevation, topographic slope, distance to the nearest river, distance to the nearest road, distance to the territory edge, and four National Land Cover classes shown relative to class ‘other’ which described all categorizes that had less than 2% of the total data. Estimates are ranked from high to low. The breeding period model included distance to the nest site.

Model: non-breeding period						
Variable	Estimate	Std. Error	CI (2.5%)	CI (97.5%)	z value	Pr(>|z|)
Intercept	-1.37	0.12	-1.61	-1.12	11.02	< 0.001
Distance to territory edge	0.87	0.01	0.85	0.88	104.47	< 0.001
Land cover: Dense bush	0.57	0.07	0.43	0.71	7.97	< 0.001
Land cover: Grassland	-0.55	0.07	-0.69	-0.40	7.40	< 0.001
Elevation	0.18	0.02	0.14	0.21	9.49	< 0.001
Distance to river	0.06	0.01	0.03	0.08	4.52	< 0.001
Slope	0.05	0.01	0.04	0.07	7.43	< 0.001
Land cover: Open bush	0.05	0.07	-0.09	0.19	0.74	0.46
Tree cover	0.03	0.01	0.02	0.05	4.33	< 0.001
Distance to road	0.01	0.01	0.00	0.03	0.82	0.41
Model: Breeding period						
Variable	Estimate	Std. Error	CI (2.5%)	CI (97.5%)	z value	Pr(>|z|)
(Intercept)	-3.01	0.44	-3.88	-2.14	6.78	< 0.001
Land cover: Dense bush	2.79	0.50	1.81	3.76	5.58	< 0.001
Land cover: Open bush	2.63	0.50	1.65	3.61	5.28	< 0.001
Land cover: Grassland	2.20	0.50	1.22	3.19	4.40	< 0.001
Distance to territory edge	2.10	0.04	2.02	2.19	47.71	< 0.001
ID: G32554	-1.98	0.10	-2.19	-1.78	18.92	< 0.001
Elevation	-1.05	0.14	-1.33	-0.77	7.37	< 0.001
ID: G32553	-0.85	0.10	-1.04	-0.65	8.47	< 0.001
Distance to nest	-0.43	0.04	-0.50	-0.35	10.89	< 0.001
Distance to river	0.40	0.04	0.32	0.48	9.47	< 0.001
Distance to road	0.29	0.03	0.23	0.34	10.14	< 0.001
Tree cover	0.08	0.03	0.02	0.13	2.77	< 0.01
Slope	0.01	0.02	-0.03	0.08	0.46	0.64

During the breeding period, habitat preference model results were similar to the non-breeding period model results, and again the top two models were averaged because they were within 4 AICc from the top model. However, during the breeding period, birds showed preference for lower elevations within their breeding home ranges. Furthermore, as expected, birds tended to be found closer to their nest sites.

The model with lowest AICc for the non-breeding period had an AUC = 0.73, and for the breeding period had an AUC = 0.89, suggesting these models had relatively good predictive power, i.e. the models’ ability to discern between presence and pseudo-absence locations was relatively strong.

## Discussion

### Home ranges

Using GPS tracking data we were able, for the first time, to describe the home range size of adult Martial Eagles. Our tracking data suggested adults held home ranges of ca. 108 km^2^. Our home ranges were similar to those of some other large eagles such as Golden Eagles (*Aquila chrysaetos*) [59]. Based on a simple circular area, our home range sizes would represent territories of ca. 6 km radius. Although our study represents the first home range estimation of this species from GPS tracking data, the results compare well with previous home range estimates based on inter-nest distances for this species [20, 60] as well as estimates from VHF radio-tagged birds [36]. For example, Tarboton and Allan [20] found inter-nest distances in the local region (KNP and Transvaal Province) of ca. 12 km (radius = 6 km). The species inter-nest distance varies considerably in different landscapes with larger inter-nest distances (ca. 19 km) in the drier regions of the Nama-Karoo and Namibia [60]. Provided that Martial Eagle inter-nest distances differ between populations possibly due to differences in habitat structure, it will be important to determine actual home range sizes in other regions if we are to determine how transferable how home ranges are to other regions.

Previous home range estimates for the species using radio tags on four individuals estimated that Martial Eagles in Kenya [36] had home ranges described by MCPs averaging 120 km^2^ (sd: 59 km^2^, range: 74–205 km^2^), with male home ranges larger than females within pairs. These home range sizes compare well with our KDE home range sizes, but poorly with our MCP home ranges. Our MCP estimates were often many times larger than our KDE estimates due to occasional movements far beyond the typical home range identified from our GPS fixes (S3 Fig). These types of large infrequent movements would unlikely be detected by conventional VHF radio tracking that have been used in other studies, and this highlights the discrepancy in accuracies between these two methods [36]. Because Martial Eagles have never been GPS tracked before, these findings are novel, however they may be expected as extra territory movements are known to occur in other raptor species and may be linked to extra pair copulations [61–63] or explorations of neighbouring territories or food patches [62, 64]. In one of these long range movements a male individual failed to return to his range, and follow up investigations suggest the bird was electrocuted in Swaziland (Fig 3). Although our sample sizes were small, the home range sizes of Martial Eagles likely represent the population given the similarities to other studies using different estimation techniques e.g. inter-nest (territory) interpolation.

Although any conclusions were severely limited by our sample size of only four breeding female birds, from three of these birds there did appear to be a pattern between home range size and breeding state, with home range estimates being lowest during and before incubation and the early chick-rearing phase. Birds also tended to make shorter trips during the breeding period. Such a finding is hardly surprising given that females of most eagle species undertake the majority of incubation and stay at the nest to care for young nestlings [26], and will therefore spend more of their time in close proximity to their nest sites during the early breeding cycle [65]. This finding was also supported by a tendency to be found closer to the nest during the breeding period. However, after the chick was older than 4 weeks, the females’ home range sizes appeared to increase presumably as they spent less time brooding and increasingly hunted within their territory to capture prey for the chick. Nest visits by our tagged birds dropped sharply after 12 weeks, supporting observations by Steyn [35]. Similar patterns in reduction of home range size during the breeding season have also been observed in Golden Eagles [66]. These results therefore emphasize the importance of studying ranging behaviour through a full breeding cycle to gain a more complete understanding of behaviour [67–69]. Furthermore, step lengths tended to increase during the dry winter months, suggesting that prey shortages during this time may have facilitated greater movement across the territory in search of prey [70].

Two individuals were population floaters, both ranging into Mozambique where they likely died due to anthropogenic causes. Individual G32516 was found caught in a hunting snare and G32551’s tag was recovered from a hunting outpost. These wide ranging behaviours were not expected for adult Marital Eagles given that a floater population has not been identified previously in the region, and it was assumed that adults likely held territories for most of their breeding lives [35]. Although our sample size is small, these behaviours are interesting, and appear to increase the risk of mortality, given that both the floater individuals as well as the long ranged movement of another individual into Swaziland resulted in non-natural mortality. Increasing our sample size of tracked adults would be useful to determine how common these kinds of behaviours are amongst adults of this species. Understanding the survival and movements of this sector of the population will likely improve our ability to understand the current population declines [39, 71]. The movements into neighbouring countries and the detected mortalities in these regions also highlights the importance of trans-boundary conservation efforts [72].

The presence of adult floaters in a declining population could be viewed as a surprising result from this study; population equilibrium theory suggests these individuals should mostly be present in a saturated breeding population where new territories are unavailable, otherwise these individuals should be expected take up vacant territories [39, 73]. We however did not detect any ousting of individuals, as regular nest checks did not detect breeding in these territories after individuals emigrated from their territories. Thus, in this declining population, the abandonment of territories and a possible increase in the floater population may signal underlying environmental limitations to breeding such as shortages of prey, mate loss, low breeding success, or limited appropriate breeding habitat in the KNP [74–77]. This is further supported by increasing home range sizes across years for two individuals; in KNP a drought was experienced during the 2014/2015 season and this may have impacted on territorial birds. For example home range sizes of Golden Eagles in the Mojave Desert are known to increase during hotter months [68]. Similarly, floaters may reject settling in these unsuitable vacant territories as the costs of holding a territory may outweigh the expected life time reproductive rate and joining the floater population could improve overall individual fitness [78]. However, an addition of floaters to the population may further impact on the breeding population through disruption via competition for more suitable territories being held by breeders, thus adding an additional stress to those individuals that choose to remain in a territory and attempt to breed [79].

### Habitat use

The habitat preferences of Martial Eagles found in this study, both in the breeding and non-breeding period indicates that the species typically preferred areas away from the territory edge, areas classified by the land cover map as dense bush rather than open bush or grasslands, and preferred areas of greater tree cover. Eagles also preferred areas with increased elevation, steeper slopes, and away from rivers and roads.

A predators habitat preference should generally relate to the preferences of their main prey items or the degree to which that habitat provides opportunities to catch prey. Riverine habitat often supports high avian biodiversity [80] and many of the Martial Eagles typical prey species, such as Galliformes and monitor lizards (e.g. *Varanus albigularis*), occupy riverine habitats [81]. Therefore it was surprising that we did not observe a tendency for increased occurrence closer to rivers but finer spatial scale river layers that map smaller drainage lines may provide better information about the species preference for this habitat. Tree cover and dense bushveld, on the other hand, were both important predictors of habitat use for Martial Eagles. Martial Eagles are known to prefer nesting in the shrubland regions of the drier Karoo regions of South Africa [27]. Dense bushveld is also more likely to carry higher primary productivity which Martial Eagles tend to prefer [82] compared to both open bushveld or grasslands. Martial Eagles use trees to hunt from, and to perch and roost on [32]. Martial Eagles may use surprise attacks as a strategy that could work better from perches and in areas with dense bush [83]. Examinations of step lengths and net square displacement plots further highlights that the species is mostly sedentary making use of short movements interspersed by longer movements, therefore likely relying on good perch spots. The importance of large trees for nesting and perching are also highlighted in the literature for Martial Eagles [25], and many other large birds of prey [84–86]. Datasets on actual tree composition in specific height classes (e.g. detailing the locations of very tall trees) may also provide better information on the species habitat preference rather than a single layer describing the density of trees over 5 m.

The reliance on tree cover is concerning as tree cover in KNP has undergone substantial change over the last half century, with some areas reducing in woody cover by up to ca. 64% [87]. These declines have been attributed largely to interactions between increasing elephant densities and frequent fires driven by historical management decisions [87, 88]. Elephants (*Loxodonta africana*) tend to impact maturing trees in the 5–9 m height range and tree fall rates in areas accessible to elephants may be up to six times higher [89]. Provided the biodiversity benefits of areas with substantial woody vegetation [90] it is plausible that increases in elephant numbers have decreased the quality of habitat or reduced nest site availability for Martial Eagles in KNP. This finding is supported by a previous analysis [17], which found that declines of Martial Eagles within KNP were highest in areas with highest elephant densities. This issue therefore clearly merits further research.

Roads are increasingly recognized to influence biotic relationships in protected areas. For instance, road effects on the localized hydrology often leads to increased woody cover along the road verge [53] and other predators have been found to spend more time within closer proximity to roads than expected as they may act as corridors for movement [91]. However, in contrast to those findings, Martial Eagles were more likely to use areas away from roads. Large eagles may be sensitive to anthropogenic disturbances such as traffic [92], however some pairs of Martial Eagles have established nests in close proximity to roads, and only 30% of roads and tracks in KNP are accessible to tourists, thus alternative explanations for the apparent avoidance of roads may be likely. For instance, animals (prey) living close to roads or open areas may have higher vigilance levels, or avoid these open areas [93, 94].

Martial Eagles avoided their territory edge and this is likely to avoid conspecific conflict, as most raptors are highly territorial and display little territory overlap with neighbours [63]. In this study two individuals for whom we recorded conspecific conflict both died from their encounter (Table 1). The Authors have also observed a number of other similar incidents involving Martial Eagles. Martial Eagles preferred areas with higher elevation and steep slopes, and although speculative, these areas may provide vantage points for intruder detection and greater visibility of prey. However it is more likely that these features may aid in flight for instance by providing orographic lift [95]. Although the distance to the territory edge is not a variable readily available to others thus reducing the generality of the model for predicting Martial Eagle habitat preference, the variable greatly improved the ability of the model to discern between pseudo-absence and presence locations (without this variable the AUC decreases by 13% points–not reported).

The habitat preference of Martial Eagles may be further explained by other variables that were not available such as prey distribution as it is known that prey availability can influence the movements of other raptors [96, 97], however because Martial Eagles can feed on a wide range of prey [35], these data sets may be challenging to collect over large spatial scales in any great detail.

### Conclusions

This study provides the first satellite-derived description of Martial Eagle ranging behaviour and habitat preference. The conservation of Martial Eagles will likely be challenging given their large ranging behaviour both when holding territories, but also when present as floaters in the population. Low-density species are more prone to stochastic events and the recovery of the population poses a considerable conservation challenge. Efforts to mitigate habitat loss e.g. tall tree loss and dense shrubland, and improving trans-boundary conservation will be important factors in the species conservation plans.

## Supporting information

S1 Fig Mapping of data used in modeling Martial Eagle habitat utilization showing the heterogeneity of the landscape in Kruger National Park. Tree cover was sourced from Sexton et al. [51], a 72 class National Land Cover (72 class NLC, from http://bgis.sanbi.org) was used to understand the preferred landscape types. A 1:50 000 river map was used to inform river importance [52]. A 90m Digital Elevation Model [50] and the derived slope were used to understand topographic influences. Roads and management tracks were provided by SANParks GIS Services and used to understand road effects.
(TIFF)

S2 Fig Net Squared Displacement (square distance between each point and the first location, plotted over time) of Martial Eagles tracked from Kruger National Park showing six individuals that remained in spatially confined areas for the majority of their tracking period and two individuals (G32516 and G32551) that roamed widely. Plots are not to the same scale due to the large variation between individuals’ movements through time.
(TIFF)

S3 Fig Minimum Convex Polygon (dashed red lines) enclosing all (100%) tracking locations of birds that held stable home ranges and the trajectory (blue lines) of those locations showing movements over the course of each individuals tracking period. (TIFF)

S1 Table A 72 class National Land Cover map (SANBI) was used to inform habitat preferences of Martial Eagles. Categories that contained < 2% of absence and presence points were collapsed into a class “other”.
(DOCX)

S2 Table Generalised linear mixed model showing how Martial Eagle hourly step lengths are affected by the breeding status (breeding vs. non-breeding period), the month of the year, and territorial behaviour. (DOCX)

S3 Table Table showing the number of GPS locations (presence points) used in the habitat preference models for each bird. Data that are used in the breeding period model are shown in bold.
(DOCX)

S4 Table Akaike Information Criteria (AICc) and associated statistics for the top five GLMMs for the non-breeding period habitat utilization of Martial Eagles in relation to tree cover (TC), National Land Cover class (LC), distance to nearest river (DRi), elevation (El), slope (Sl), distance to the territory edge (Ed), and distance to nearest road (DRo). And AICc associated statistics for the top five GLMs for the breeding period habitat utilization of Martial Eagles including distance to the nearest nest site (Ne).
(DOCX)

S1 File Data used in modelling the habitat preference of Martial Eagles during the breeding period. (CSV)

S2 File Data used in modelling the habitat preference of Martial Eagles during the non-breeding period. (CSV)
