# Supplementary figures and images for: Ranging behaviour and habitat preferences of the Martial Eagle: Implications for the conservation of a declining apex predator
Source: PLoS One. 2017 Mar 17;12(3):e0173956. doi: 10.1371/journal.pone.0173956 (PMC5357022; doi:10.1371/journal.pone.0173956)

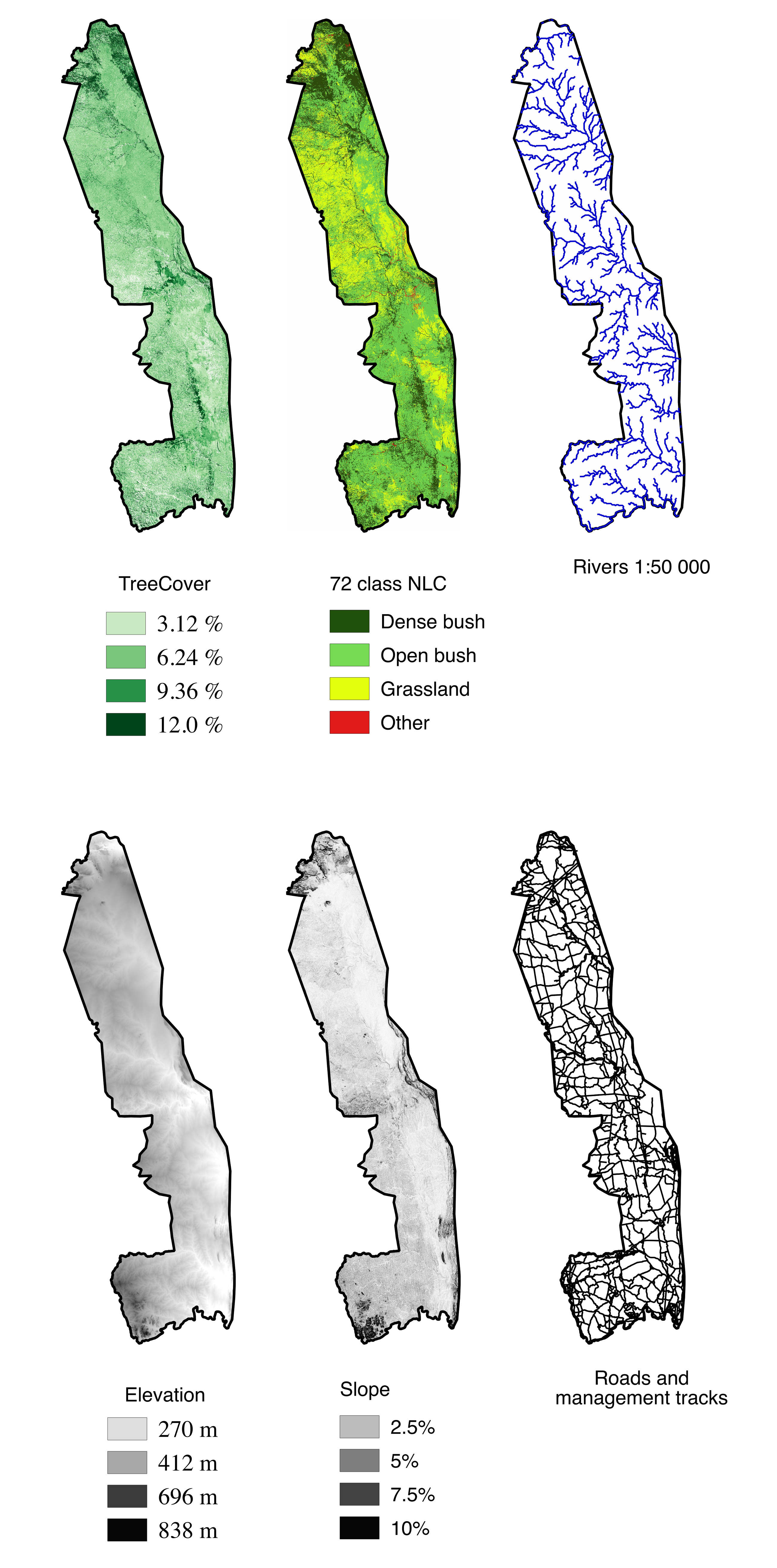

Supplement: S1 Fig — Tree cover was sourced from Sexton et al. [51], a 72 class National Land Cover (72 class NLC, from http://bgis.sanbi.org) was used to understand the preferred landscape types. A 1:50 000 river map was used to inform river importance [52]. A 90m Digital Elevation Model [50] and the derived slope were used to understand topographic influences. Roads and management tracks were provided by SANParks GIS Services and used to understand road effects. (TIFF) [file pone.0173956.s001.tiff]

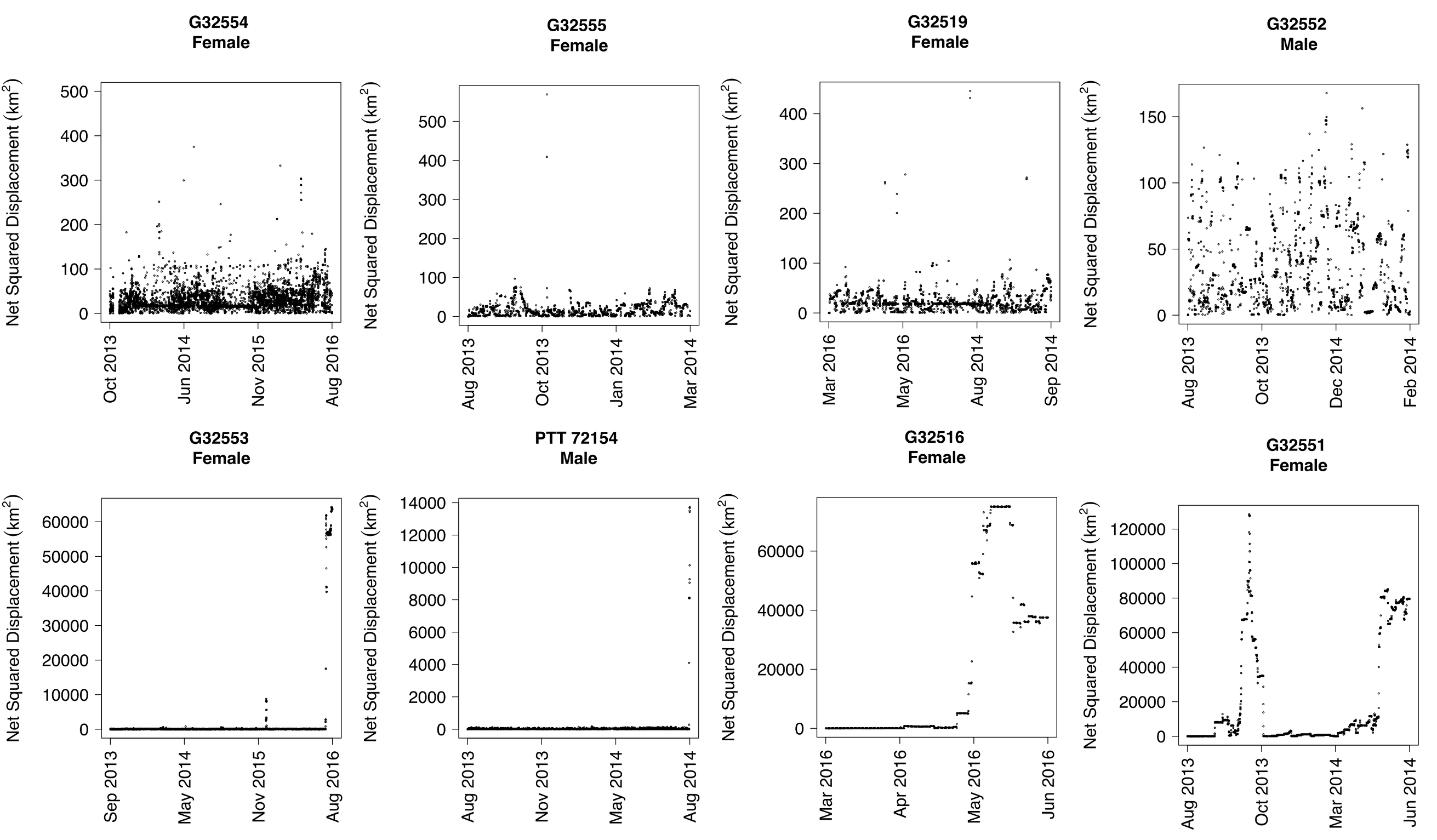

Supplement: S2 Fig — Plots are not to the same scale due to the large variation between individuals’ movements through time. (TIFF) [file pone.0173956.s002.tiff]

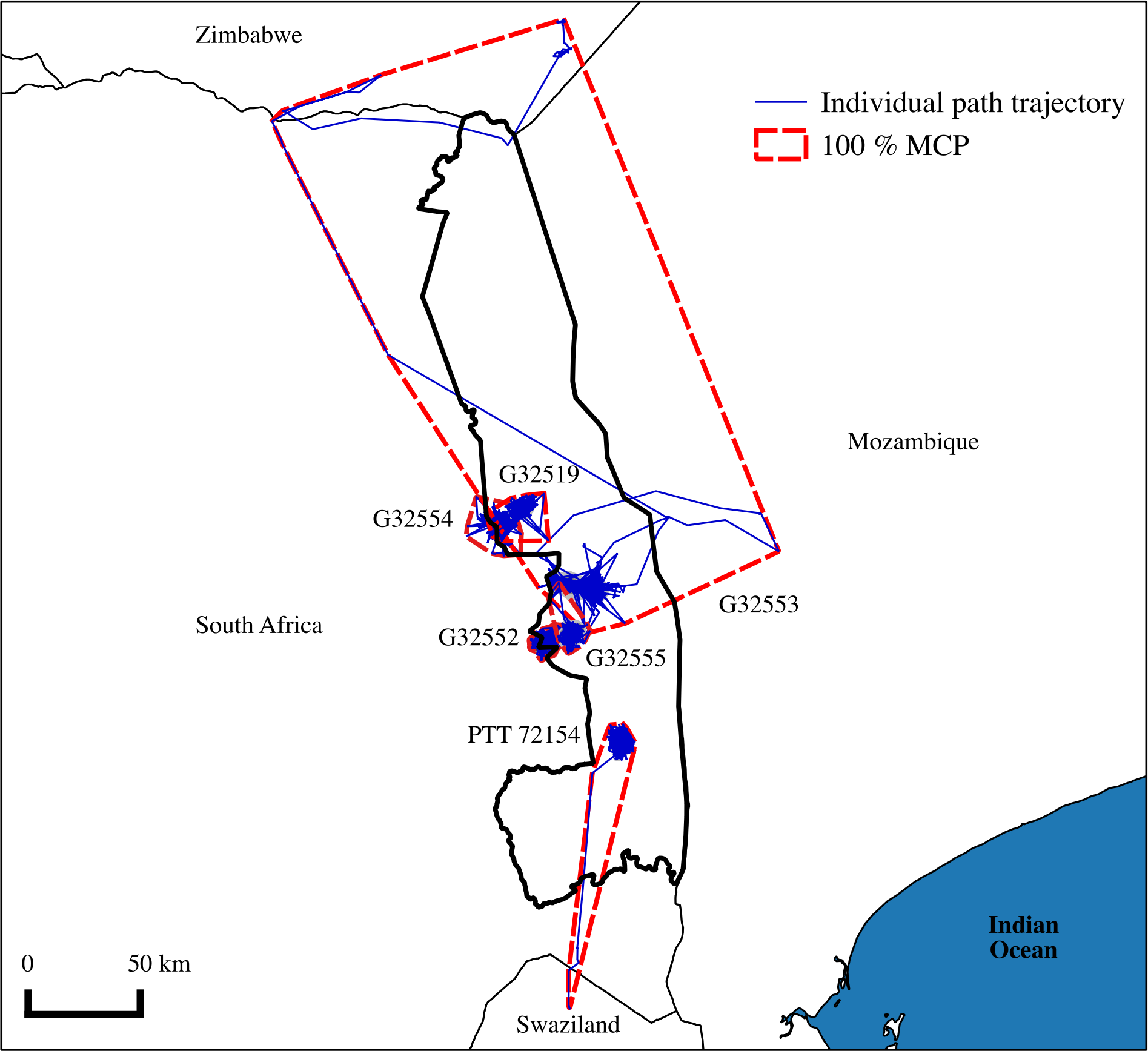

Supplement: S3 Fig — (TIFF) [file pone.0173956.s003.tiff]
