# Supplementary material for: Ranging behaviour and habitat preferences of the Martial Eagle: Implications for the conservation of a declining apex predator
Source: PLoS One. 2017 Mar 17;12(3):e0173956. doi: 10.1371/journal.pone.0173956 (PMC5357022; doi:10.1371/journal.pone.0173956)
Supplement: S1 Table — Categories that contained < 2% of absence and presence points were collapsed into a class “other”. (DOCX) [file pone.0173956.s004.docx]

Table S1. A 72 class National Land Cover map (SANBI) was used to inform habitat preferences of Martial Eagles. Categories that contained < 2% of absence and presence points were collapsed into a class “other”.

| Class | Description | Presence points | Absence points |
| --- | --- | --- | --- |
| 36 | Mines – semi-bare | 1 | 12 |
| 53 | Urban smallholding (dense trees / bush) | 1 |  |
| 58 | Urban sports and golf (open trees / bush) | 1 | 47 |
| 69 | Urban built-up (dense trees / bush) | 2 | 17 |
| 72 | Urban built-up (bare) | 2 | 2 |
| 57 | Urban sports and golf (dense trees / bush) | 3 | 120 |
| 48 | Urban residential (dense trees / bush) | 4 |  |
| 70 | Urban built-up (open trees / bush) | 5 | 9 |
| 40 | Erosion | 11 | 40 |
| 35 | Mines – bare | 13 | 237 |
| 41 | Bare / non vegetated | 15 | 87 |
| 2 | Water permanent | 18 | 69 |
| 9 | Low shrubland | 154 | 290 |
| 7 | Grassland | 1826 | 9017 |
| 5 | Thicket /dense bush | 6319 | 11775 |
| 6 | Woodland /open bush | 15329 | 49376 |
| 1 | Water seasonal |  | 3 |
| 3 | Wetlands |  |  |
| 4 | Indigenous forest |  |  |
| 8 | Shrubland fynbos |  |  |
| 10 | Cultivated comm fields (high) |  |  |
| 11 | Cultivated comm fields (med) |  |  |
| 12 | Cultivated comm fields (low) |  |  |
| 13 | Cultivated comm pivots (high) |  |  |
| 14 | Cultivated comm pivots (med) |  |  |
| 15 | Cultivated comm pivots (low) |  |  |
| 16 | Cultivated orchards (high) |  |  |
| 17 | Cultivated orchards (med) |  |  |
| 18 | Cultivated orchards (low) |  |  |
| 19 | Cultivated vines (high) |  |  |
| 20 | Cultivated vines (med) |  |  |
| 21 | Cultivated vines (low) |  |  |
| 22 | Cultivated permanent pineapple |  |  |
| 23 | Cultivated subsistence (high) |  |  |
| 24 | Cultivated subsistence (med) |  |  |
| 25 | Cultivated subsistence (low) |  |  |
| 26 | Cultivated cane pivot - crop |  |  |
| 27 | Cultivated cane pivot - fallow |  |  |
| 28 | Cultivated cane commercial - crop |  |  |
| 29 | Cultivated cane commercial - fallow |  |  |
| 30 | Cultivated cane emerging - crop |  |  |
| 31 | Cultivated cane emerging - fallow |  |  |
| 32 | Plantations / woodlots mature |  |  |
| 33 | Plantations / woodlots young |  |  |
| 34 | Plantations / woodlots clearfelled |  |  |
| 37 | Mines - water seasonal |  | 2 |
| 38 | Mines – water permanent |  | 2 |
| 39 | Mine buildings |  |  |
| 42 | Urban commercial |  |  |
| 43 | Urban industrial |  |  |
| 44 | Urban informal (dense trees / bush) |  |  |
| 45 | Urban informal (open trees / bush) |  |  |
| 46 | Urban informal (low veg / grass) |  |  |
| 47 | Urban informal (bare) |  |  |
| 49 | Urban residential (open trees / bush) |  |  |
| 50 | Urban residential (low veg / grass) |  |  |
| 51 | Urban residential (bare) |  |  |
| 52 | Urban school and sports |  |  |
| 54 | Urban smallholding (open trees / bush) |  |  |
| 55 | Urban smallholding (low veg / grass) |  |  |
| 56 | Urban smallholding (bare) |  |  |
| 59 | Urban sports and golf (low veg / grass) |  | 5 |
| 60 | Urban sports and golf (bare) |  | 1 |
| 61 | Urban township (dense trees / bush) |  |  |
| 62 | Urban township (open trees / bush) |  |  |
| 63 | Urban township (low veg / grass) |  |  |
| 64 | Urban township (bare) |  |  |
| 65 | Urban village (dense trees / bush) |  |  |
| 66 | Urban village (open trees / bush) |  |  |
| 67 | Urban village (low veg / grass) |  |  |
| 68 | Urban village (bare) |  |  |
| 71 | Urban built-up (low veg / grass) |  |  |
