# Supplementary material for: Ranging behaviour and habitat preferences of the Martial Eagle: Implications for the conservation of a declining apex predator
Source: PLoS One. 2017 Mar 17;12(3):e0173956. doi: 10.1371/journal.pone.0173956 (PMC5357022; doi:10.1371/journal.pone.0173956)
Supplement: S2 Table — (DOCX) [file pone.0173956.s005.docx]

Table S2. Generalized linear mixed model results showing the estimated effects of breeding period, month and territoriality on step length.

| Variable | Estimate | Std. Error | CI (2.5 %) | CI (97.5 %) | z value | Pr(>\|z\|) |
| --- | --- | --- | --- | --- | --- | --- |
| (Intercept) | 0.89 | 0.13 | 0.64 | 1.14 | 7.00 | < 0.001 |
| Breeding: Yes | -0.31 | 0.07 | -0.44 | -0.18 | 4.78 | < 0.001 |
| Month: 2 | 0.11 | 0.08 | -0.04 | 0.26 | 1.44 | 0.15 |
| Month: 3 | 0.09 | 0.07 | -0.05 | 0.24 | 1.26 | 0.21 |
| Month: 4 | 0.47 | 0.08 | 0.32 | 0.62 | 6.19 | < 0.001 |
| Month: 5 | 0.78 | 0.08 | 0.62 | 0.94 | 9.65 | < 0.001 |
| Month: 6 | 0.79 | 0.09 | 0.62 | 0.96 | 9.27 | < 0.001 |
| Month: 7 | 0.52 | 0.09 | 0.34 | 0.69 | 5.92 | < 0.001 |
| Month: 8 | 0.85 | 0.07 | 0.70 | 1.00 | 11.35 | < 0.001 |
| Month: 9 | 0.35 | 0.07 | 0.21 | 0.49 | 4.87 | < 0.001 |
| Month: 10 | 0.70 | 0.07 | 0.55 | 0.84 | 9.64 | < 0.001 |
| Month: 11 | 0.29 | 0.07 | 0.14 | 0.43 | 3.88 | < 0.001 |
| Month: 12 | 0.00 | 0.07 | -0.14 | 0.14 | 0.01 | 0.99 |
| Territorial: Yes | -0.03 | 0.11 | -0.55 | 0.25 | 0.27 | 0.79 |
