# Supplementary material for: Ranging behaviour and habitat preferences of the Martial Eagle: Implications for the conservation of a declining apex predator
Source: PLoS One. 2017 Mar 17;12(3):e0173956. doi: 10.1371/journal.pone.0173956 (PMC5357022; doi:10.1371/journal.pone.0173956)
Supplement: S3 Table — Data that are used in the breeding period model are shown in bold. (DOCX) [file pone.0173956.s006.docx]

Table S3. Table showing the number of GPS locations (presence points) used in the habitat preference models for each bird. Data that are used in the breeding period model are shown in bold.

|  | Year: 2013 | |  |  |  |  |  |  |  |  |  |  |
| --- | --- | --- | --- | --- | --- | --- | --- | --- | --- | --- | --- | --- |
| ID | Jan | Feb | Mar | Apr | May | Jun | Jul | Aug | Sep | Oct | Nov | Dec |
| G32554 |  |  |  |  |  |  |  |  |  | 220 | 112 | 478 |
| G32553 |  |  |  |  |  |  |  | 72 | 401 | 414 | 366 | 407 |
| G32552 |  |  |  |  |  |  |  | 120 | 453 | 456 | 451 | 471 |
| G32555 |  |  |  |  |  |  |  |  | 156 | 479 | 407 | 476 |
| PTT 72154 |  |  |  |  |  |  | 19 | 425 | 463 | 480 | 443 | 480 |
|  |  |  |  |  |  |  |  |  |  |  |  |  |
|  | Year: 2014 | |  |  |  |  |  |  |  |  |  |  |
| ID | Jan | Feb | Mar | Apr | May | Jun | Jul | Aug | Sep | Oct | Nov | Dec |
| G32554 | 441 | 405 | 406 | 192 | **98** | **55** | 115 | 315 | 358 | 437 | 427 | 423 |
| G32553 | 458 | 419 | 451 | **404** | **358** | **335** | **319** | 399 | 406 | 457 | 446 | 484 |
| G32552 | 459 | 94 |  |  |  |  |  |  |  |  |  |  |
| G32555 | 467 | 250 |  |  |  |  |  |  |  |  |  |  |
| PTT 72154 | 490 | 434 | 458 | 232 | 427 | 414 | 421 | 280 |  |  |  |  |
|  |  |  |  |  |  |  |  |  |  |  |  |  |
|  | Year: 2015 | |  |  |  |  |  |  |  |  |  |  |
| ID | Jan | Feb | Mar | Apr | May | Jun | Jul | Aug | Sep | Oct | Nov | Dec |
| G32554 | 456 | 372 | 416 | **389** | **323** | **345** | **272** | 216 | 317 | 336 | 335 | 412 |
| G32553 | 415 | 332 | 405 | 355 | 390 | 330 | 336 | 378 | 344 | 415 | 332 | 405 |
|  |  |  |  |  |  |  |  |  |  |  |  |  |
|  | Year: 2016 | |  |  |  |  |  |  |  |  |  |  |
|  |  |  |  |  |  |  |  |  |  |  |  |  |
| ID |  |  |  |  |  |  |  |  |  |  |  |  |
| G32554 | 378 | 373 | 347 | 394 | 256 | 286 | 145 | 211 | 158 |  |  |  |
| G32553 | 444 | 418 | 445 | 398 | 303 | 313 | 228 | 259 |  |  |  |  |
| G32519 |  |  | 191 | 456 | **428** | **411** | **423** | 428 | 310 |  |  |  |
