# Supplementary material for: Ranging behaviour and habitat preferences of the Martial Eagle: Implications for the conservation of a declining apex predator
Source: PLoS One. 2017 Mar 17;12(3):e0173956. doi: 10.1371/journal.pone.0173956 (PMC5357022; doi:10.1371/journal.pone.0173956)
Supplement: S4 Table — And AICc associated statistics for the top five GLMs for the breeding period habitat utilization of Martial Eagles including distance to the nearest nest site (Ne). (DOCX) [file pone.0173956.s007.docx]

Table S4. Akaike Information Criteria (AICc) and associated statistics for the top five GLMMs for the non-breeding period habitat utilization of Martial Eagles in relation to tree cover (TC), National Land Cover class (LC), distance to nearest river (DRi), elevation (El), slope (Sl), distance to the territory edge (Ed), and distance to nearest road (DRo). And AICc associated statistics for the top five GLMs for the breeding period habitat utilization of Martial Eagles including distance to the nearest nest site (Ne).

| Model: non breeding period | Df | AICc | ΔAICc | Weight |
| --- | --- | --- | --- | --- |
| El + LC + Sl + DRo + DRi + TC + Ed | 11 | 128925.57 | 0.00 | 0.56 |
| LC +Sl + DRo + DRi + TC + Ed | 10 | 128926.06 | 0.49 | 0.44 |
| El + LC + Sl + DRo + TC + Ed | 10 | 128942.19 | 16.62 | < 0.001 |
| El + LC + Sl + DRi + Ed | 9 | 128942.32 | 16.74 | < 0.001 |
| El + LC + Sl + DRo + DRi + Ed | 10 | 128942.51 | 16.93 | < 0.001 |
|  |  |  |  |  |
| Model: breeding period | Df | AICc | ΔAICc | Weight |
| El + LC + DRo + DRi + TC + Ed + Ne + ID | 12 | 11201.38 | 0.00 | 0.60 |
| El + LC + Sl + DRo + DRi + TC + Ed + Ne + ID | 13 | 11202.47 | 1.09 | 0.35 |
| El + LC + DRo + DRi + Ed + Ne + ID | 11 | 11206.81 | 5.43 | 0.04 |
| El + LC + Sl + DRo + DRi + Ed + Ne + ID | 12 | 11208.21 | 6.83 | 0.02 |
| LC + DRo + DRi + TC + Ed + Ne + ID | 11 | 11276.15 | 74.77 | < 0.001 |
